# Natural killer cells control a T-helper 1 response in patients with Behçet's disease

**DOI:** 10.1186/ar3005

**Published:** 2010-05-11

**Authors:** Yukie Yamaguchi, Hayato Takahashi, Takashi Satoh, Yuka Okazaki, Nobuhisa Mizuki, Kazuo Takahashi, Zenro Ikezawa, Masataka Kuwana

**Affiliations:** 1 Division of Rheumatology, Department of Internal Medicine, Keio University School of Medicine, 35 Shinanomachi, Shinjuku-ku, Tokyo 160-8582, Japan; 2 Department of Environmental Immuno-Dermatology, Yokohama City University Graduate School of Medicine, 3-9 Fukuura, Kanazawa-ku, Yokohama 236-0004, Japan; 3 Department of Dermatology, Keio University School of Medicine, 35 Shinanomachi, Shinjuku-ku, Tokyo 160-8582, Japan; 4 Department of Ophthalmology, Yokohama City University Graduate School of Medicine, 3-9 Fukuura, Kanazawa-ku, Yokohama 236-0004, Japan

## Abstract

**Introduction:**

Behçet's disease (BD) is a multisystem inflammatory disorder, in which a T-helper 1 (Th1)-polarized immune response plays a major role in the pathogenic process. We evaluated the regulatory role of natural killer (NK) cells in Th1-biased immune responses in patients with BD.

**Methods:**

We studied 47 patients with BD, including 10 with active disease (aBD) and 37 with inactive disease (iBD), and 29 healthy controls. The activation status and cytotoxic activity of NK cells were examined by flow cytometry. The levels of mRNAs for immune modulatory and cytotoxic molecules in NK cells were determined by quantitative PCR. The IL-12 signal strength in NK cells was determined by assessing the phosphorylation state of its downstream component, signal transducer and activator of transduction 4, by immunoblotting. Finally, NK cells' ability to modulate the Th1 response was evaluated by co-culturing NK cells and T cells without cell contact.

**Results:**

CD69^+^-activated NK cells were significantly increased in aBD compared with iBD or control samples, although their cytotoxic activities were similar. The iBD NK cells showed downregulated IL-12 receptor β_2 _mRNA levels compared with aBD or control NK cells. The increased IL-13 expression was detected in a subset of BD patients: most of them had iBD. The IL-13 expression level in iBD patients was significantly higher than the level in controls, but was not statistically different compared with the level in aBD patients. The gene expression profile in iBD patients was consistent with the NK type 2 phenotype, and the shift to NK type 2 was associated with disease remission. NK cells from iBD patients showed impaired IL-12-induced signal transducer and activator of transduction 4 phosphorylation. Finally, iBD, but not control, NK cells suppressed IFNγ expression by aBD-derived CD4^+ ^T cells *in vitro*.

**Conclusions:**

NK cells may control disease flare/remission in BD patients via NK type 2-mediated modulation of the Th1 response.

## Introduction

Behçet's disease (BD) is a multisystem inflammatory disorder characterized by recurrent attacks of uveitis, genital ulcers, oral aphtoid lesions, and skin lesions such as erythema nodosum [1]. The etiology of BD remains unclear, but previous studies on the circulating CD4^+ ^T cells and affected lesions of BD patients with active disease showed elevated levels of T-helper 1 (Th1) cytokines, such as IFNγ and IL-12, indicating that a Th1-polarized immune response plays a major role in the pathogenic process [2-4]. In addition, we recently reported that cytotoxic lymphocytes, including CD8^+ ^and γδ T cells, are also involved in the pathogenesis of BD via their cytotoxic activity [5,6]. Natural killer (NK) cells are another lymphocyte subset with cytotoxic activity, but their reported numbers and cytotoxic activity in both circulation and BD-associated lesions have been inconsistent [7-9].

NK cells have long been regarded as an essential component of innate immunity, based on their nonspecific cytotoxic activity against virus-infected and tumor cells [10]. Recent evidence, however, indicates that NK cells also regulate innate and acquired immune responses through their secretion of soluble factors and/or cell-cell contact [11]. Recently, a classification of NK cells into two functional subsets based on their expression profiles of cytokines and cytokine receptors has gained wide acceptance [12]: NK type 1 (NK1) cells mainly produce IFNγ and IL-10, and express high levels of IL-12 receptor β_2 _(IL-12Rβ_2_); while NK type 2 (NK2) cells produce IL-5 and/or IL-13, and express low levels of IL-12Rβ_2_. This NK1/NK2 paradigm has been shown to control pathogenic Th1-biased or Th2-biased immune response in several human immune-mediated diseases, such as multiple sclerosis [13], asthma [14], and pemphigus vulgaris [15].

In the present study, we investigated the potential regulatory functions of NK cells in the Th1-biased environment of BD by evaluating their activation status, gene expression profiles, and functional properties in association with the disease status.

## Materials and methods

### Patients and controls

We studied 47 patients with BD (19 men and 28 women, aged 47.3 ± 17.6 years) who fulfilled the criteria proposed by an International Study Group [16]. Twenty-nine healthy individuals (14 men and 15 women, aged 38.2 ± 12.3 years) provided control samples.

The BD of the patients was classified as active (aBD) in 10 cases and inactive (iBD) in 37 cases at the time of blood sampling. Active disease was defined as flare of characteristic BD symptoms, including severe skin, mucosal, and/or ocular involvement that required introduction or increase of systemic corticosteroids (≥ 0.5 mg/kg), cyclosporine, and/or infliximab [6]. Five patients who had aBD at their first examination were re-evaluated after their BD-related symptoms resolved.

All samples were obtained after the patients and control subjects gave their written informed consent, approved by the International Review Boards of Keio University and Yokohama City University.

### HLA-B51 typing

The presence or absence of HLA-B51 was determined by PCR of the genomic DNA using sequence-specific primers and sequence-based typing [17].

### Cell preparations

Peripheral blood mononuclear cells (PBMCs) were isolated from heparinized venous blood by Lymphoprep (Fresenius Kabi Norge AS, Oslo, Norway) density-gradient centrifugation. NK cells were purified by the MACS cell isolation system (Miltenyi Biotec, Bergisch Gladbach, Germany) as CD14^-^CD3^-^CD56^+ ^cells [15]. Namely, the CD14^+ ^cells and CD3^+ ^cells were depleted from PBMCs by incubation with anti-CD14 and anti-CD3 mAb-coupled magnetic beads, and then the CD56^+ ^cells were positively selected by incubation with anti-CD56 mAb-coupled magnetic beads, according to the manufacturer's protocol. The sorted fraction contained >99.6 ± 0.2% CD56^+ ^cells, and contamination with CD3^+ ^cells was <0.3 ± 0.3%. In some experiments, T cells were also isolated as CD56^-^CD3^+ ^cells using the MACS cell isolation system.

### Activated status of natural killer cells

PBMCs were incubated with the following combination of fluorescently labeled mAbs: anti-CD56-fluorescein isothiocyanate, anti-CD69-phycoerythrin-cyanin 5.1, and anti-CD3-allophycocyanin (Beckman-Coulter, Fullerton, CA, USA). Fluorescent cell staining was detected by a FACSCalibur^® ^flow cytometer (Becton Dickinson, San Jose, CA, USA) using CellQuest™ software. Appropriate fluorescently labeled isotype-matched mAbs to irrelevant antigens were used in all analyses. The proportion of activated NK cells was assessed from the cells expressing CD69, an early activation marker of lymphocytes [18], within the CD56^+^CD3^- ^NK cell fraction.

### Cytotoxic activity

The nonspecific cytotoxic activity of NK cells was quantified by a flow cytometry-based assay using NKTEST^® ^(Orpegen Pharma, Heidelberg, Germany). Briefly, K562 target cells pre-stained with a lipophilic green fluorescent membrane dye were mixed with freshly isolated effector PBMCs at an effector-to-target ratio of 25:1 and were incubated for 2 hours at 37° C. Dead cells were detected by incubation with a DNA staining solution and subsequent analysis on a flow cytometer. The specific cytotoxicity (%) was determined by subtracting the proportion of dead cells in the mock-treated sample from the proportion in the sample pre-treated with effector cells.

### Expression of genes associated with NK1/NK2 phenotype and cytotoxicity

The total RNA was extracted from MACS-sorted NK cells using an RNeasy^® ^mini kit (Qiagen, Hilden, Germany), and was subjected to oligo (dT)-primed reverse transcription to generate first-strand cDNA. The cDNA equivalent to 5 ng total RNA was subjected to semiquantitative PCR to detect IL-12Rβ2, IFNγ, IL-5, IL-10, IL-13, perforin, granzyme B, and glyceraldehyde-3-phosphate dehydrogenase (GAPDH), using specific primer sets as described elsewhere [15]. The PCR products were fractionated on agarose gels and visualized by ethidium bromide staining. The intensity of individual bands was semiquantitatively analyzed using the Image/J^® ^software [19]. The relative expression level of individual genes was normalized to the expression of GAPDH.

The mRNA expression of selected genes was further evaluated using a quantitative Taqman^® ^real-time PCR system (Applied Biosystems, Foster City, CA, USA). All primers and probes were purchased from Applied Biosystems. The gene expression was standardized based on serial amounts of cDNA prepared from a healthy donor's PBMCs that were stimulated with phorbol 12-myristate-13-acetate and ionomycin [15]. The relative expression levels of individual genes were normalized to the expression level of GAPDH.

### Phosphorylation status of signal transducer and activator of transduction 4

The phosphorylated signal transducer and activator of transduction 4 (Stat4) and total Stat4 in IL-12-stimulated NK cells was detected by immunoblots as previously described [15]. The antibodies used were rabbit anti-phosphorylated-Stat4 polyclonal antibodies (Zymed Laboratories, South San Francisco, CA, USA) and rabbit anti-Stat4 polyclonal antibodies (Santa Cruz Biotechnology, Santa Cruz, CA, USA). The intensity of individual bands with the expected molecular sizes was semiquantitatively analyzed using the image/J^® ^software. The phosphorylation status of Stat4 was expressed as the ratio of the intensity of phosphorylated Stat4 to that of total Stat4.

### IFNγ expression in CD4+ T cells co-cultured with natural killer cells

The capacity of NK cells to modulate the expression of IFNγ by T cells was evaluated using a cell-contact-free co-culture system. Briefly, MACS-sorted T cells (2 × 10^6^) obtained from aBD patients were cultured in RPMI1640 supplemented with 7.5% low IgG fetal bovine serum (HyClone, South Logan, UT, USA) with or without sorted NK cells (5 × 10^5^) prepared from iBD patients or healthy controls, applied to the upper chamber of an insert separated by a 0.4 μm pore-size membrane (BD Biosciences, San Jose, CA, USA) on 12-well plastic plates, for 12 hours at 37°C. Leukocyte Activation Cocktail^® ^(5 μl/well; BD Biosciences) was added at the initiation of the culture. The T cells were then fixed and permeabilized using an Intracellular Cytokine Staining Kit Human^® ^(BD Biosciences), and were subsequently stained with anti-IFNγ-phycoerythrin (BD Biosciences) and anti-CD4-phycoerythrin-cyanin 5.1 (Beckman-Coulter), according to the manufacturer's protocols. The appropriate fluorescently labeled control antibodies were used to define the background Immunofluorescence of the cells. Finally, the cells were subjected to flow cytometry, and the IFNγ expression level on the gated CD4^+ ^T cells was calculated as a mean fluorescence intensity using CellQuest™ software. The relative IFNγ expression was calculated as the ratio of IFNγ expression by CD4^+ ^T cells cultured with NK cells to the expression by CD4^+ ^T cells cultured alone.

### Statistical analysis

All results are expressed as the mean ± standard deviation. Statistical comparisons between two groups were performed using the Mann-Whitney *U *test. Serial measurements were statistically evaluated by the Wilcoxon *t *test.

## Results

### Clinical features of Behçet's disease patients

Of 47 patients with BD, 100%, 61%, 96%, and 28% had had oral ulcer, uveitis, skin lesion, and genital ulcer, respectively, during the course of the disease. Only a small proportion of the patients had history of intestinal (6%), vascular (11%), and neurological (6%) involvement. HLA-B51 was detected in 28 patients (60%). Treatment at the time of blood sampling included colchicine (n = 13), low-dose prednisolone (n = 3), cyclosporine (n = 2), etanercept (n = 1), colchicine and low-dose prednisolone (n = 4), low-dose prednisolone and methotrexate (n = 1), colchicine, low-dose prednisolone and cyclosporine (n = 1), colchicine, low-dose prednisolone and azathioprine (n = 1), and colchicine, low-dose prednisolone and infliximab (n = 1). Twenty patients (42%) received no treatment.

Ten patients (21%) were classified as having aBD at the time of blood sampling, based on a major uveitis attack (n = 7), intestinal flare with a minor uveitis attack (n = 2), or exacerbation of mucocutaneous symptoms with high fever (n = 1). None of the aBD patients had concomitant flare of vascular or neurological involvement. There was no difference in the frequency of HLA-B51 or treatment regimens between aBD and iBD. Seven patients with uveitis attack were treated with infliximab (n = 4), cyclosporine (n = 1), or an increased dosage of prednisolone in combination with cyclosporine (n = 2), resulting in resolution of symptoms within 3 months. Two patients with intestinal flare were treated with infliximab, resulting in resolution of all intestinal symptoms within 3 months. The mucocutaneous flare in the remaining patient was improved by high-dose prednisolone in combination with an increase in the dosage of cyclosporine.

### Activation status of natural killer cells

**Figure 1:**
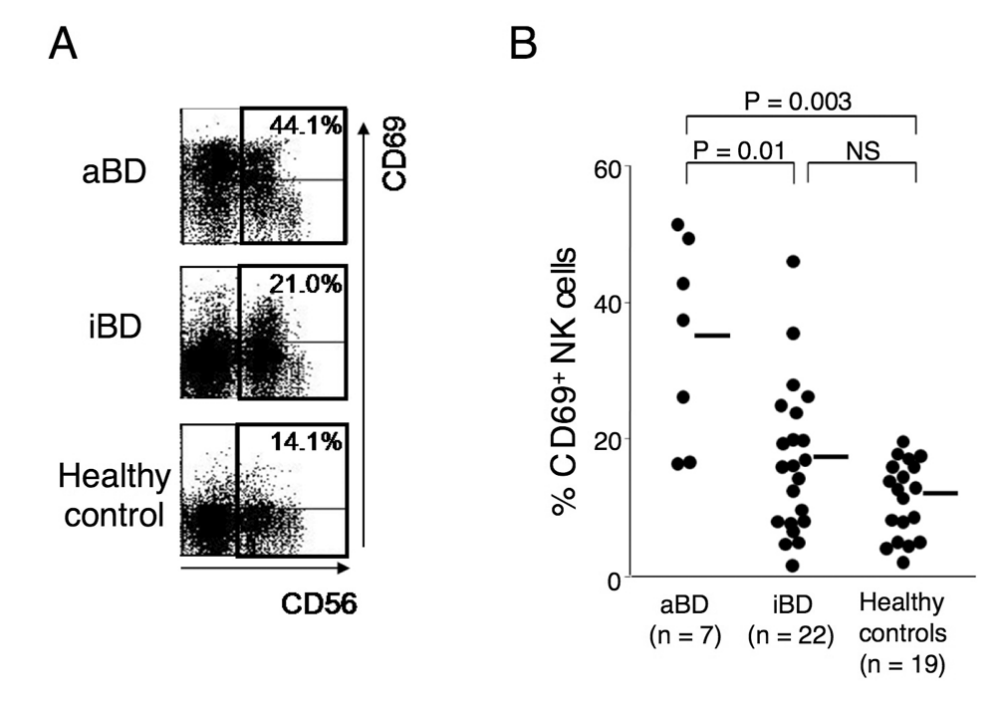
**Activation status of natural killer cells in Behçet's disease**. Proportion of activated natural killer (NK) cells in active Behçet's disease (aBD) patients, inactive Behçet's disease (iBD) patients, and healthy controls. **(a) **Representative dot-plot analysis for the expression of CD69 and CD56 in the gated CD3^- ^lymphocytes from a patient with aBD, a patient with iBD, and a healthy control. The numbers indicate the proportion of CD69^+^-activated cells in total NK cells. **(b) **Proportion of CD69^+^-activated NK cells in seven aBD patients, 22 iBD patients, and 19 healthy controls. Horizontal bars, mean values. NS, not significant.

We determined the activation status of the circulating NK cells in seven patients with aBD, 22 patients with iBD, and 19 healthy controls by examining the CD69 expression on the NK cells. As shown in Figure 1, the proportion of CD69^+^-activated NK cells was significantly greater in the aBD patients than in the iBD patients or healthy controls (*P *= 0.01 and *P *= 0.003, respectively). There was a trend toward an increased proportion of activated NK cells in the iBD patients compared with in healthy controls, but the difference did not reach statistical significance (*P *= 0.1). These findings indicate that *in vivo *activation of circulating NK cells is observed in patients with aBD, but is not remarkable in those with iBD.

### Cytotoxic activity of natural killer cells

There was no difference in the nonspecific cytotoxic activity among the NK cells from three patients with aBD, 10 patients with iBD, and 13 healthy controls (14.9 ± 10.3%, 14.3 ± 5.4%, and 14.4 ± 7.4%, respectively).

### Gene expression profiles of natural killer cells

NK cells freshly isolated from aBD patients, iBD patients, and healthy controls were first subjected to semiquantitative PCR to measure the expression of genes associated with NK1/NK2 differentiation and cytotoxicity, including those encoding IL-12Rβ2, IL-5, IL-10, IL-13, IFNγ, perforin, and granzyme B. Of these molecules, the mRNA levels of IL-12Rβ2, perforin, and granzyme B were significantly lower, and that of IL-13 was significantly higher, in the iBD patients than in the aBD patients or healthy controls (*P *<0.05 for all comparisons) (data not shown). No IL-5 expression was detected in any of the samples from BD patients or healthy controls, and there was no statistically significant difference in the expression level of IL-10 or IFNγ.

**Figure 2:**
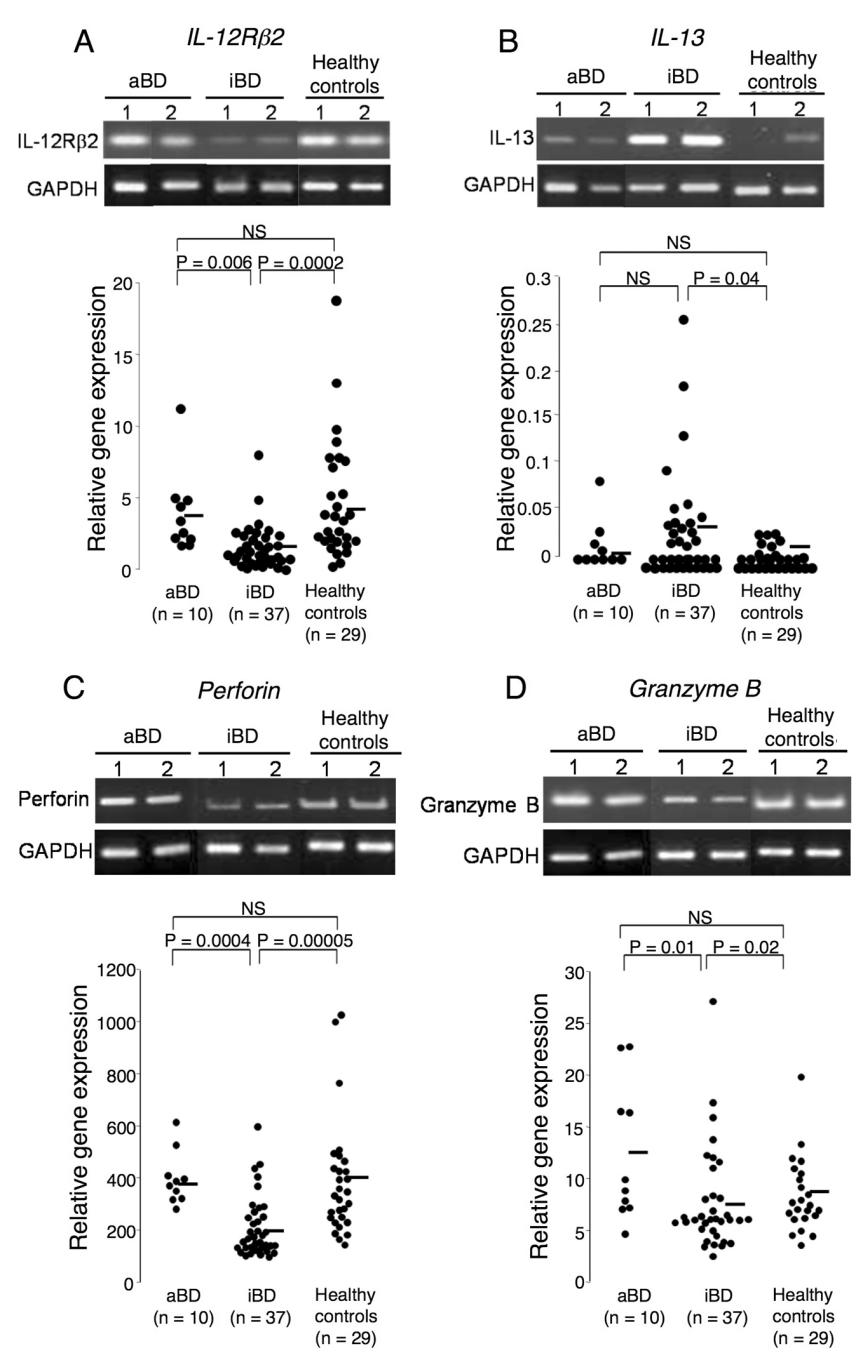
**Gene expression levels of natural killer cells in Behçet's disease**. Gene expression levels of **(a) **interleukin-12 receptor β_2 _(IL-12Rβ2), **(b) **IL-13, **(c) **perforin, and **(d) **granzyme B in natural killer (NK) cells from active Behçet's disease patients (aBD), inactive Behçet's disease (iBD) patients, and healthy controls. The IL-12Rβ2, IL-13, perforin, and granzyme B expression levels in the NK cells were evaluated using semiquantitative PCR: two representative images each from aBD patients, iBD patients, and healthy controls are shown in the upper portion of each panel. The relative gene expression levels were further determined by quantitative Taqman^® ^real-time PCR in 10 aBD patients, 37 iBD patients, and 29 healthy controls: results are shown in the lower portion of each panel. Horizontal bars, mean values. GADPH, glyceraldehyde-3-phosphate dehydrogenase; NS, not significant.

To confirm the results obtained by semiquantitative PCR, the gene expression levels of IL-12Rβ2, IL-13, perforin, and granzyme B were further evaluated by quantitative TaqMan^® ^real-time PCR (Figure 2). The IL-12Rβ2 expression was significantly lower in the iBD patients than in the aBD patients or healthy controls (*P *= 0.006 and *P *= 0.0002, respectively). The increased IL-13 expression was detected in a subset of patients with BD: most of them had iBD. The IL-13 expression level in iBD patients was significantly higher than the level in the healthy controls (*P *= 0.04), and tended to be higher than the level in aBD patients (*P *= 0.2). Interestingly, differences in IL-12Rβ2 and IL-13 levels were not detectable between the aBD patients and healthy controls.

These findings indicated that the NK cells from iBD patients have a gene expression profile compatible with NK2; that is, upregulated IL-13 and downregulated IL-12Rβ2. On the other hand, the expression levels of perforin and granzyme B were significantly lower in the iBD patients than in the aBD patients or healthy controls (*P *<0.02 for all comparisons), while these expression levels were similar between these aBD patients and healthy controls.

### Serial gene expression analysis of natural killer cells

**Figure 3:**
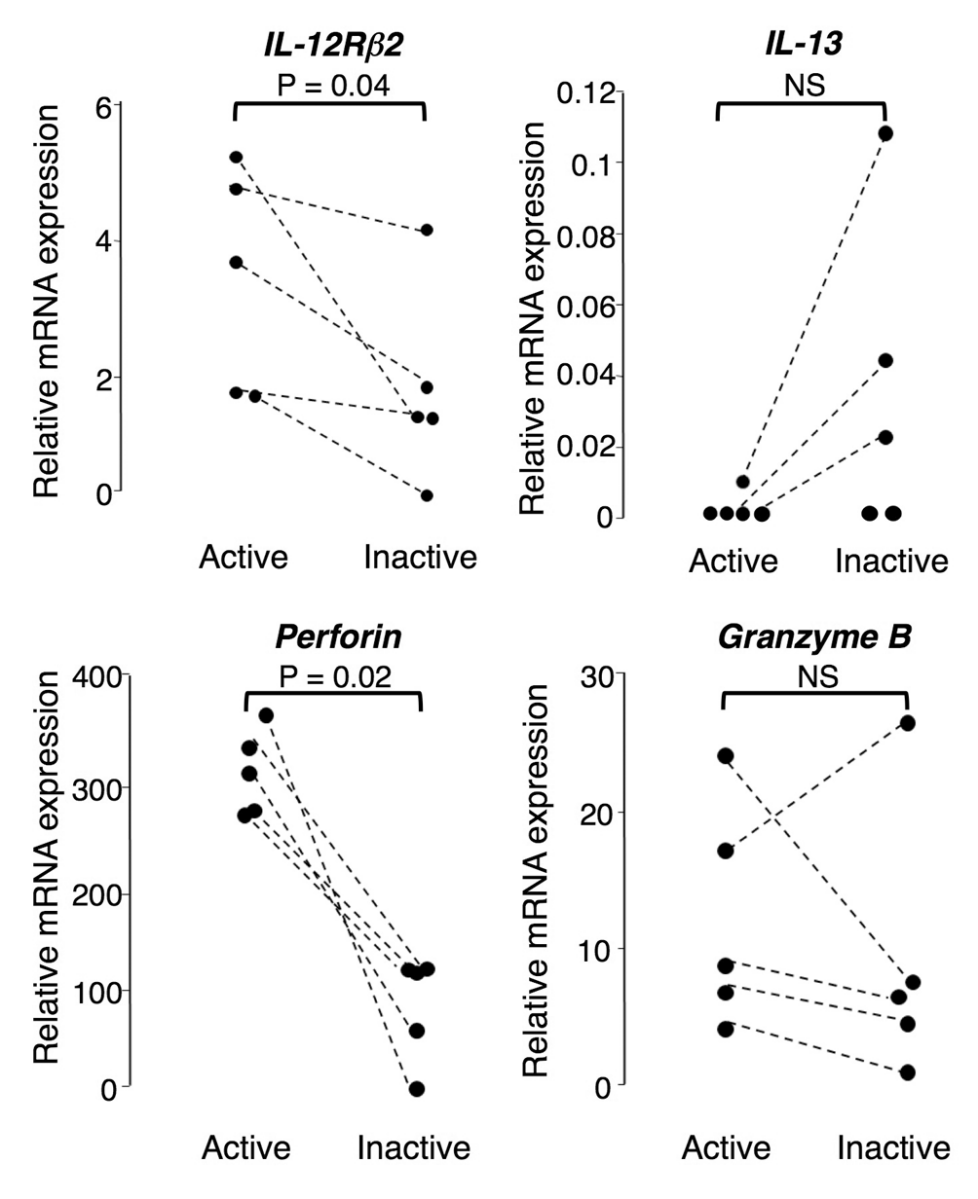
**Serial gene expression measurements of natural killer cells in active Behçet's disease**. Serial gene expression measurements of interleukin-12 receptor β_2 _(IL-12Rβ2), IL-13, perforin, and granzyme B in natural killer (NK) cells from patients with active Behçet's disease at the first evaluation. The relative gene expression levels in NK cells were determined by quantitative PCR in samples obtained at the time of active disease and at a follow-up visit during remission. NS, not significant.

For five aBD patients, additional blood samples were available when their BD symptoms were resolved after the introduction of infliximab (n = 2) or cyclosporine (n = 1), or of an increased dosage of prednisolone in combination with cyclosporine (n = 2). The gene expression level of IL-12Rβ2 was reduced in all five patients as the disease status became quiescent (*P *= 0.04) (Figure 3). IL-13 expression became detectable in three of the patients, and the change was borderline but did not reach a statistical significance (*P *= 0.05). These results strongly suggest that an NK2 shift was associated with disease remission. In addition, the expression level of perforin was reduced when the patients' disease status changed to remission (*P *= 0.02).

### Impaired IL-12 signaling in natural killer cells from inactive Behçet's disease patients

**Figure 4:**
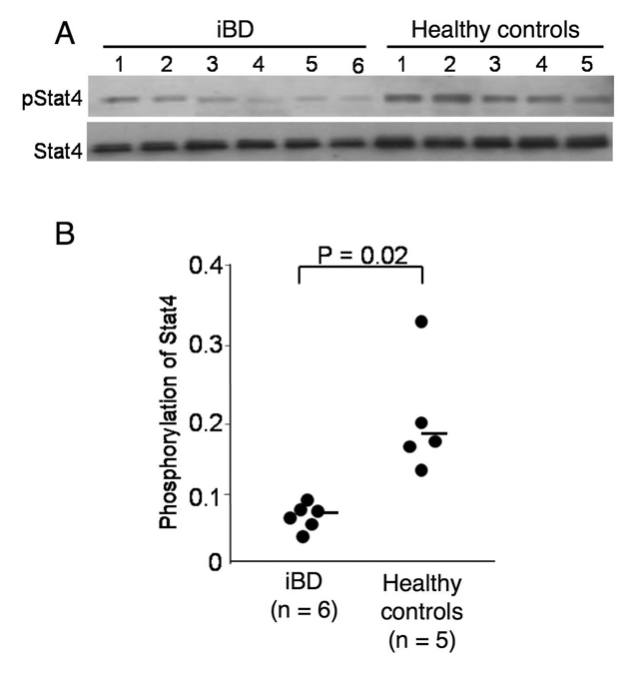
**IL-12 signaling of natural killer cells in inactive Behçet's disease patients**. IL-12-induced signal transducer and activator of transduction 4 (Stat4) phosphorylation in natural killer (NK) cells from inactive Behçet's disease (iBD) patients and healthy controls. **(a) **NK cells from six iBD patients and five healthy controls were stimulated with IL-12, and were subjected to immunoblotting for the detection of phosphorylated Stat4 (pStat4) and total Stat4. **(b) **Phosphorylation status of Stat4, which is expressed as the ratio of the intensity of pStat4 to that of total Stat4, in NK cells from six iBD patients and five healthy controls. Horizontal bars, mean values.

The downregulated IL-12Rβ_2 _gene expression observed in the NK cells from iBD patients could lead to impaired IL-12 signaling. To test this possibility, the phosphorylation status of Stat4, which is a downstream component of the IL-12 signaling pathway [20], was evaluated in the NK cells from six iBD patients and five healthy controls. As shown in Figure 4, the IL-12-induced Stat4 phosphorylation was significantly lower in the iBD patients than in the healthy controls (*P *= 0.02).

### Capacity of natural killer cells from inactive Behçet's disease patients to suppress IFNγ expression by Th1 cells

**Figure 5:**
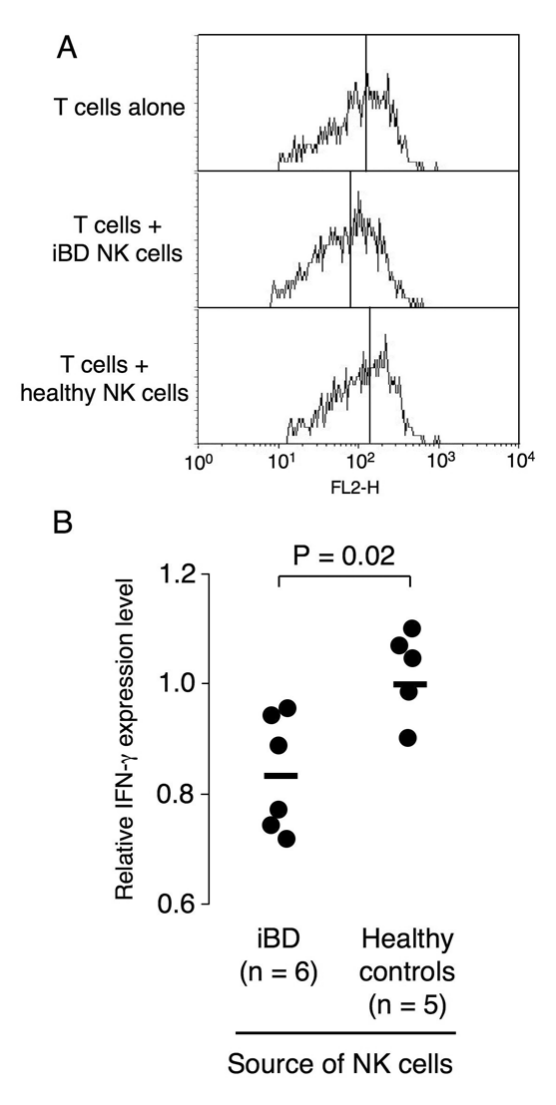
**Natural killer cell suppression of IFNγ expression by T-helper 1 cells in inactive Behçet's disease**. Suppression of IFNγ expression in T-helper 1 (Th1) cells by cell-contact-free co-culture with natural killer (NK) type 2 cells from inactive Behçet's disease (iBD) patients. T cells from active Behçet's disease patients were cultured alone or in combination with NK cells from healthy controls or iBD patients, and the IFNγ expression level in the CD4^+ ^T cells was evaluated using flow cytometry. **(a) **Representative histogram plots showing expression of IFNγ on gated CD4^+ ^T cells that were cultured alone, or with NK cells from an iBD patient or a healthy control. Vertical line in each histogram indicates the median. **(b) **IFNγ expression levels in Th1 cells cultured with the NK cells from six iBD patients or from five healthy controls. Relative IFNγ expression level calculated as the ratio of IFNγ expression by CD4^+ ^T cells cultured with NK cells to the expression by CD4^+ ^T cells cultured alone. Horizontal bars, mean values.

The NK2 bias observed in patients with iBD led us to speculate that NK cells play a role in controlling the pathogenic Th1 response in BD patients. To evaluate this hypothesis, the NK cells from iBD patients or healthy controls were co-cultured with Th1 cells derived from aBD patients in a cell-contact-free system. The intracellular IFNγ expression in the gated CD4^+ ^T cells was then analyzed using flow cytometry (Figure 5). We found that the level of IFNγ expressed by Th1 cells was reduced after their co-culture with the NK cells derived from iBD patients. In fact, the relative IFNγ expression level was significantly lower in the Th1 cells co-cultured with iBD patients' NK cells compared with the level in those co-cultured with healthy controls' NK cells (*P *= 0.02). These findings suggest that the NK2 cells from iBD patients can suppress the Th1 response in aBD patients without cognate cell-cell contact.

## Discussion

The present study has shown that the NK cells were phenotypically altered in BD patients, especially those in inactive disease status. Features of the circulating NK cells in iBD patients included: downregulated gene expression of IL-12Rβ_2_; upregulated gene expression of IL-13 in a subset of the patients; downregulated perforin and granzyme B gene levels; and impaired IL-12-induced Stat4 phosphorylation. Upregulated IL-13 and downregulated IL-12Rβ2 observed in NK cells from iBD patients were compatible with the NK2 phenotype. A serial NK phenotype analysis in aBD patients supported the association between NK2 bias and inactive disease status. Furthermore, NK2 cells obtained from iBD patients directly suppressed the IFNγ expression of Th1 cells derived from aBD patients *in vitro*. These findings together suggest that the NK1/NK2 balance modulates disease flare/remission in BD patients by controlling the pathogenic Th1 response. This situation is analogous to multiple sclerosis, another Th1-mediated disease, in which NK2 bias is associated with disease remission [13].

A major limitation of this study is the small number of patients analyzed, especially those with aBD. During 2 years of the study period, only 10 patients with aBD were enrolled in two major university hospitals in the Tokyo metropolitan area. In addition, there was a limited chance of obtaining peripheral blood samples from patients with aBD, because such patients required immediate introduction of treatment. Further multicenter studies involving a large number of patients with aBD are necessary to confirm our findings. Another limitation is the difficulty in classifying BD patients into those with active disease and those with inactive disease. We used a strict definition to select patients with aBD: flare of characteristic BD symptoms that required introduction of the intensive treatment, such as high-dose corticosteroids, cyclosporine, and infliximab. Patients with mild mucocutaneous manifestations or minor uveitis attack, which did not require intensive therapy, were therefore classified as having iBD. This clinical heterogeneity in the iBD subset may result in variability in the gene and protein expression profiles. Additional analysis according to individual clinical manifestations and/or treatment regimens would clarify these issues, but again the number of patients enrolled was too small to conduct subanalysis. Finally, we should recognize that a series of experiments involved only a subset of the patients and controls, which potentially bias the results.

Our results suggest that the NK2 cells in iBD patients can suppress the Th1 response through at least two distinct mechanisms. First, the NK2 cells in iBD patients were intrinsically hyporesponsive to IL-12 due to their downregulated expression of IL-12Rβ_2 _and impaired IL-12 signaling, resulting in deficient IFNγ production even in the Th1 environment. Second, the NK2 cells from iBD patients actively suppressed IFNγ expression in aBD-derived Th1 cells. A similar inhibitory effect of human NK2 cells on the production of IFNγ by T cells was also reported for healthy individuals' NK cells that were induced to express the NK2 phenotype [13], and for NK2 cells obtained from multiple sclerosis patients in remission [21]. Taken together, the NK cells and T cells - two major IFNγ producers - were deficient in IFNγ production in the NK2-biased immune environment observed in iBD patients.

How the NK2 cells from iBD patients suppress the IFNγ expression in Th1 cells, however, remains unclear. One potential soluble mediator in our cell-contact-free culture system is IL-13, a typical T-helper 2 cytokine that inhibits Th1 responses *in vitro *and *in vivo *[22,23], although upregulated IL-13 expression was detected only in one-third of the iBD patients. In addition, this IL-13-mediated inhibitory effect is reported to occur predominantly through the modulation of antigen-presenting cells rather than as a direct effect on T cells [22]. Additional soluble factors secreted from NK2 cells are likely to be involved in this regulation, but the NK cells from iBD patients did not express IL-5, which plays a primary role in Th1 inhibition in multiple sclerosis patients in remission [13]. Furthermore, it has been reported that NK cells modulate Th1 responses also by interacting directly with T cells, B cells, and dendritic cells though cognate cell-cell contact [24,25].

Perforin and granzyme B, major cytoplasmic granule toxins, were downregulated in the NK cells from patients with iBD. Interestingly, this gene expression profile is analogous to that of the NK cells in patients with active pemphigus vulgaris, who also show NK2 bias [15]. This phenomenon could be explained by the reduced IL-12Rβ_2 _expression and impaired IL-12 signaling, but the cytotoxic activity was the same among the NK cells of iBD patients, aBD patients, and healthy controls. The reason for this inconsistency is unknown, but the cytotoxic activity of NK cells might be regulated by more complicated mechanisms, involving a balance between activating and inhibitory NK receptors, as well as the expression of the ligands for death receptors on target cells [26].

In aBD patients, the proportion of activated NK cells in the circulation was markedly increased. This is reasonable because IL-12 can activate NK cells in the Th1 environment, even though the nonspecific cytotoxic activity and gene expression profiles were similar between the NK cells from aBD patients and healthy controls. These activated NK cells would migrate to sites of inflammation and contribute to the ongoing tissue damage in aBD patients, but this appears to be just a bystander effect of the Th1 environment of aBD.

## Conclusions

The present study is the first demonstrating a novel regulatory role for NK cells in the pathogenic process of BD. Our results have suggested that NK cells are actively involved in the induction and maintenance of disease remission in BD patients, through NK2 polarization. Future studies aimed at elucidating the mechanisms that control the NK1/NK2 paradigm in BD patients may be useful for developing new NK cell-targeted therapeutic strategies for BD.

## Abbreviations

aBD: active Behçet's disease; BD: Behçet's disease; GAPDH: glyceraldehyde-3-phosphate dehydrogenase; iBD: inactive Behçet's disease; IFN: interferon; IL: interleukin; IL-12Rβ_2_: interleukin-12 receptor β_2_; mAb: monoclonal antibody; NK: natural killer; NK1: natural killer type 1; NK2: natural killer type 2; PBMC: peripheral blood mononuclear cell; PCR: polymerase-chain reaction; Stat4: signal transducer and activator of transduction 4; Th1: T-helper 1.

## Competing interests

The authors declare that they have no competing interests.

## Authors' contributions

YY performed the acquisition of data, and analysis and interpretation of the data, and wrote the manuscript. HT made a substantial contribution to the acquisition of data. TS and YO performed the acquisition of data. NM, KT, and ZI provided peripheral blood samples and clinical information, and performed analysis of the data. MK designed the experiments, performed data analysis and interpretation, and wrote the manuscript. All authors read and approved the final manuscript.
